# Spatial Characteristics and Factor Analysis of Pollution Emission from Heavy-Duty Diesel Trucks in the Beijing–Tianjin–Hebei Region, China

**DOI:** 10.3390/ijerph16244973

**Published:** 2019-12-06

**Authors:** Beibei Zhang, Sheng Wu, Shifen Cheng, Feng Lu, Peng Peng

**Affiliations:** 1 The Academy of Digital China, Fuzhou University, Fuzhou 350002, China; zhangbeibily@163.com (B.Z.); ws0110@163.com (S.W.); luf@lreis.ac.cn (F.L.); 2 State Key Laboratory of Resources and Environmental Information System, Institute of Geographic Sciences and Natural Resources Research, Chinese Academy of Sciences, Beijing 100101, China; pengp@lreis.ac.cn; 3 University of Chinese Academy of Sciences, Beijing 100049, China

**Keywords:** heavy-duty diesel trucks, socioeconomic factors, spatial autocorrelation characteristic, spatial econometric model, geographical detector technique

## Abstract

Heavy-duty diesel trucks (HDDTs) contribute significantly to NO_X_ and particulate matter (PM) pollution. Although existing studies have emphasized that HDDTs play a dominant role in vehicular pollution, the spatial distribution pattern of HDDT emissions and their related socioeconomic factors are unclear. To fill this research gap, this study investigates the spatial distribution pattern and spatial autocorrelation characteristics of NO_X_, PM, and SO_2_ emissions from HDDTs in 200 districts and counties of the Beijing–Tianjin–Hebei (BTH) region. We used the spatial lag model to calculate the significances and directions of the pollutants from HDDTs and their related socioeconomic factors, namely, per capita GDP, population density, urbanization rate, and proportions of secondary and tertiary industries. Then, the geographical detector technique was applied to quantify the strengths of the significant socioeconomic factors of HDDT emissions. The results show that (1) NO_X_, PM, and SO_2_ pollutants emitted by HDDTs in the BTH region have spatial heterogeneity, i.e., low in the north and high in the east and south. (2) The pollutants from HDDTs in the BTH region have significant spatial autocorrelation characteristics. The spatial dependence effect was obvious; for every 1% increase in the HDDT emissions in the surrounding districts and counties, the local HDDT emissions increased by 0.39%. (3) Related factors analysis showed that the proportion of tertiary industries had a significant negative correlation, whereas the proportion of secondary industries and urbanization rate had significant positive correlations with HDDT emissions. Population density and per capita GDP did not pass the significance test. (4) The order of effect intensities of the significant socioeconomic factors was proportion of tertiary industry > proportion of secondary industry > urbanization rate. This study guides scientific decision making for pollution control of HDDTs in the BTH region.

## 1. Introduction

The annual increase in the number of vehicles in China has enhanced the severity of environmental pollution caused by vehicle exhaust [1,2,3,4,5]. Motor vehicle emissions have become major sources of air pollution in megacities in China [6] such as Beijing, Shanghai, and Guangzhou. Among different categories of vehicles, heavy-duty diesel trucks (HDDTs) have attracted widespread attention because of their significant contribution to NO_X_ and particulate matter (PM) pollution [5,7,8,9,10,11]. The annual report for 2018 by China’s Motor Vehicle Environmental Management [12] shows that HDDTs were responsible for approximately 59.9% and 53.4% of the total PM and NO_X_ emissions, respectively, from on-road vehicles. Therefore, the state has formulated a series of policies and regulations for pollution control of HDDTs. For example, in January 2019, the Ministry of Ecology and Environment of China issued the Action Plan for Combating Pollution from Diesel Trucks [13] which listed the Beijing–Tianjin–Hebei (BTH) region as a key area. It clearly proposed to strengthen the control of high emissions from diesel trucks, strengthen the supervision of diesel trucks during severe fog and haze, and accelerate the complete adoption of Euro VI gasoline and diesel in the BTH region and its surrounding cities. Although these policies effectively reduce HDDT emissions, a thorough understanding of the spatial distribution pattern and related influencing factors of HDDT emissions are essential to formulate scientific and reasonable emission reduction measures. Therefore, it is necessary to perform research on HDDT emissions in the BTH region.

Several detailed studies have been performed on vehicle emissions. Regarding the spatial characteristics of vehicle emissions, existing studies focus on the spatial distribution pattern and spatial autocorrelation characteristics of vehicle emissions. They allocate vehicle emissions to the regular grid [14,15,16,17,18] in grid-based analysis of pollution data, or visually analyze the spatial distribution trend of vehicle pollutants based on county [19], prefecture [20], and administrative divisions. A few studies have used on-board emission measurements to measure the emissions and their associated factors from vehicles under actual operating conditions [21]. In addition, a large number of studies have used spatial autocorrelation methods [22], such as Global Moran’s I and Local Moran’s I, to analyze the spatial dependence effect of vehicle emissions and use clustering analysis, including high–low value clustering [22], hot spot analysis [23], fuzzy c-means algorithm clustering [24], and k-means method [23], to explore the spatial clustering characteristics of regional vehicle emissions. However, most existing studies regard all types of motor vehicles uniformly. Although the dominant contribution of HDDTs to haze pollutants, which mainly includes NO_X_, PM, and SO_2_ pollutants, has been emphasized by various studies, the spatial distribution pattern and spatial agglomeration characteristics of HDDT emissions remain unclear.

In a study involving related factors analysis of motor vehicle emissions, Requia et al. [25] selected GDP, population density, road network length, urbanization rate, and other socioeconomic indicators as covariates to explore the correlation between motor vehicle emissions and cardiorespiratory diseases. Tuia et al. [26] used the spatial distribution data on population density and urbanization rate to distribute vehicle emissions. Requia et al. [23] depicted the correlation between vehicle emissions and socioeconomic factors including GDP, population, urbanization rate, road network length, human development index, and distance from the state capital by using the ordinary least squares model (OLS). The results showed that there were significant positive correlations between vehicle emissions and socioeconomic factors. However, as the main mode of road transport for medium- and long-distance bulk cargo [10,27], HDDTs undertake the transportation of goods and raw materials for manufacturing, steel, and other industries as well as logistics and express delivery. Heavy-duty diesel trucks are closely related to industrial structures. Current researches often neglect the impact of industrial structure indicators such as proportions of secondary industries and tertiary industries on motor vehicle emissions. It is necessary to comprehensively explore the impact of secondary and tertiary industries on HDDT emissions. In addition, the traditional OLS model neglects the spatial effect and produces biased and inconsistent estimation results. The pollutants emitted by motor vehicles have an obvious diffusion effect, and the air pollution from adjacent cities may have a spatial dependence effect. Compared with traditional estimation methods, a spatial econometric model can help researchers to explore whether regional environmental performance depends on the characteristics of adjacent areas [28] thus compensating for the lack of spatial dependence of classical linear models. It shows better performance in determining whether there is a significant correlation between pollutants and socioeconomic factors and the direction of the significant correlation [29,30]. Therefore, this study chose two spatial econometric models, the spatial lag model (SLM) and spatial error model (SEM), to explore the significant related factors of HDDT emissions.

After determining the significant related factors of HDDT emissions, quantifying their effect intensities can assist in further identifying the core factors affecting HDDT emissions. In recent years, Wang and Xu [31] established a geographical detector technique which is widely used in the study of related geographical influencing factors. For example, Zhou et al. [29] used the geographical detector technique to determine the strengths of association between PM_2.5_ concentration and socioeconomic factors, such as industrial dust, proportion of secondary industries, population density, road density, and per capita GDP, and found that industrial dust was the primary influencing factor for PM_2.5_ concentration. Liu and Yang [32] used the geographical detector technique to reveal the driving factors behind the urbanization of counties in China. The results showed that the main influencing factors of urbanization vary across different regions. However, the geographical detector technique is seldom used to quantitatively determine the effect of related factors on vehicle emissions in existing studies. Therefore, this technique was introduced in this study to compare the effect intensities of the significant related factors of HDDT emissions to provide policy makers with targeted policy recommendations.

Based on the above background, in order to solve the problem of unclear spatial characteristics of pollutants and unclear mechanisms of the related influencing factors of HDDTs in the BTH region, this study explored the spatial distribution pattern and spatial autocorrelation characteristics of pollutants from HDDTs in 200 districts and counties in the BTH region. We also analyzed the related influencing factors that cause these spatial characteristics from the perspective of social economy and use the spatial econometric model to calculate the significances and directions of the related factors. The geographical detector technique was used to compare the strengths of the significant related factors of HDDT emissions. This research was aimed at guiding the formulation of a policy for the coordinated development of the BTH region and effective control of diesel truck pollution control. The results can provide a basis for scientific and effective decision making for controlling HDDT emissions in the BTH region.

## 2. Study Area and Data

### 2.1. Study Area

**Figure 1:**
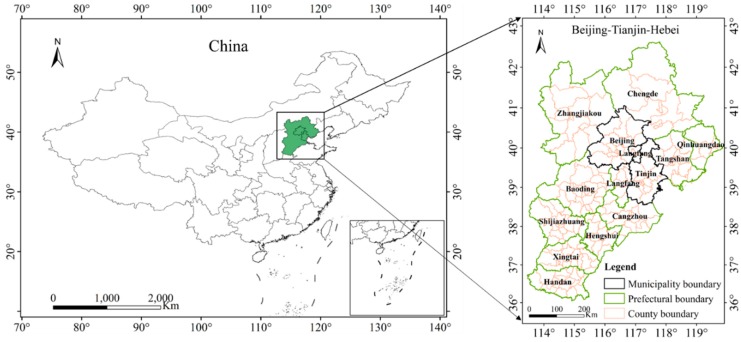
Map of the study area.

The BTH region located in the northern part of China is an important economic area in China. It includes two municipalities directly under the central government in Beijing and Tianjin as well as 11 prefecture-level cities such as Chengde, Qinhuangdao, Tangshan, Langfang, Baoding, Cangzhou, Shijiazhuang, Hengshui, Xingtai, and Handan in Hebei Province as shown in Figure 1. In 2018, the permanent population of the BTH region was 110 million, with a total GDP of 8.5 trillion yuan which accounted for 9.44% of the GDP of the entire country. The BTH region accounted for 11.7% of the total number of motor vehicles in China [33]. In recent years, fog and haze have been frequent in this region. This has strengthened the demand for coordinated governance of environmental pollution in the BTH region which has become a national strategy. Therefore, it is of great significance to regard BTH as the research area of interest.

### 2.2. Data Acquisition and Management

The data used in this paper were divided into two categories: data of pollutants from HDDT emissions and data of related socioeconomic factors. We obtained the traffic activity data and detailed specifications of HDDTs from 15 April 2018 to 15 May 2018 in the BTH region from the open data interface provided by the National Road Freight Vehicle Public Supervision and Service Platform [34] to construct the emission inventory of HDDTs for each road segment [35]. The traffic activity data included sampling time (data sampling frequency of 1 s), geographical location (longitude and latitude), vehicle identification, and actual driving speed. According to statistics, the number of vehicle trajectory records per five minutes is approximately 1.39 million, and the amount of data per five minutes reaches 40 GB. Using the map matching technology, the driving trajectories of the HDDTs were matched to the road network, and then the road network was divided to form 262,706 road segments. The average speed for each road segment can be obtained by averaging the actual driving speeds of the HDDTs for each road segment [36,37]. The detailed specifications of the HDDTs include vehicle identification, tonnage level, and emission standards. According to the Technical Guide for the Preparation of Air Pollutant Emission Inventory of Road Vehicles [38], the emission standards of HDDTs were divided into Pre-Euro, Euro I, Euro II, Euro III, Euro IV, Euro V, and Euro VI, and the tonnage level was divided into 5 categories: 12–14 t, 14–20 t, 20–28 t, 28–32 t, and >32 t. Dynamic traffic activity data and static specification information can be associated using the vehicle identification. The partial detailed data are shown in Table 1. 

With regard to the related factors of HDDT pollution, five explanatory variables were selected, namely, per capita GDP, population density, urbanization rate, secondary industry ratio, and tertiary industry ratio. The data were obtained from the Beijing Regional Statistical Yearbook (2018) [39], Tianjin Statistical Yearbook (2018) [40], and Hebei Economic Yearbook (2018) [41]. Table 2 shows the descriptive statistics of each explanatory variable, symbol predictions based on existing research results, and Global Moran’s I values. All explanatory variables had significant spatial autocorrelation characteristics. In the data processing stage of the correlation analysis, the pollution emission data (dependent variables) of the HDDTs and data pertaining to the related socioeconomic factors (independent variables) were logarithmically processed to bring the data closer to the normal distribution and eliminate the heteroscedasticity of the regression model. In data processing of the geographical detector technique, the natural discontinuity method was used to transform independent variables from numerical values to type values.

## 3. Methods

**Figure 2:**
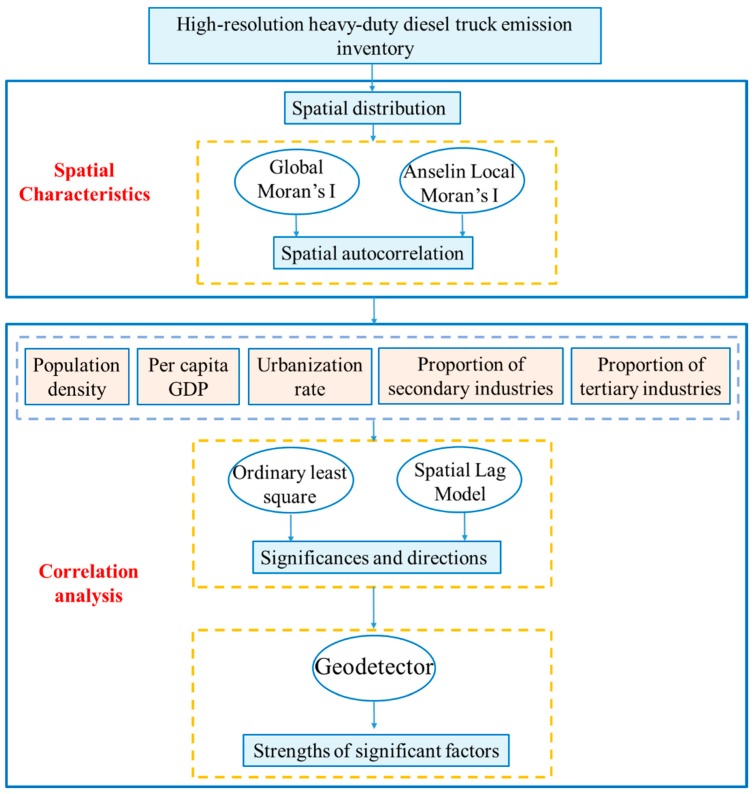
Technical method of spatial characteristics and correlation analysis for heavy-duty diesel truck emissions.

The main objective of this study was to clarify the spatial distribution characteristics of NO_X_, PM, and SO_2_ emissions from HDDTs at the district and county levels in the BTH region and to explore the interaction mechanisms between related socioeconomic factors and HDDT emissions. The overall framework is shown in Figure 2. Firstly, the spatial distribution pattern and spatial autocorrelation characteristics of NO_X_, PM, and SO_2_ emissions from HDDTs in the BTH region are explored. Secondly, regression analysis was used to explore the significances and directions of socioeconomic factors of HDDT emissions. The geographical detector technique was used to explore the strengths of the significant related factors. Finally, based on the experimental results, the spatial distribution characteristics of HDDTs and their interaction with socioeconomic factors were analyzed, which provides a scientific and reasonable basis for the government to formulate targeted emission reduction measures for HDDTs.

### 3.1. Construction of Pollutant Emission Inventory for HDDTs 

According to the idea of modeling for a single road segment to modeling for the district and county, this study calculated the emissions of NO_X_, PM, and SO_2_ from HDDTs in districts and counties in the BTH region. First, based on the COPERT V (Computer Programme to Calculate Emissions from Road Transport) model, we calculated the NO_X_, PM, and SO_2_ emissions of a single road segment according to Equation (1).

(1) $$E_{p,i,t}=\underset{m,n}{\sum }EF_{p,m,n,v,i}\times L_{i}\times 10^{-3}$$

where $E_{p,i,t}$ represents the emission in kg/5 min of pollutant p from HDDTs in road segment i at time interval t. $EF_{p,m,n,v,i}$ denotes the emission factor for pollutant p from HDDTs with emission standard n and tonnage level m at speed v in g/km. $L_{i}$ represents the length of the road segment i in km.

Secondly, according to the administrative boundaries of the BTH region, the emissions in each road segment in each district and county were counted, and the NO_X_, PM, and SO_2_ emissions of HDDTs in 200 districts and counties were obtained. Using the administrative area of each district and county, the total emissions of NO_X_, PM, and SO_2_ of HDDTs per unit area of each district and county were calculated as follows:

(2) $$E_{p,c}=\frac{\sum_{t}E_{p,i,t}}{A_{c}},\;i\in c$$

where $E_{p,c}$ represents the emission of pollutant p from HDDTs per unit area of the district and county c. $A_{c}$ denotes the area of the district and county c. i∈c refers to the road segment i within the administrative boundaries of the district and county c.

Table 3 lists the detailed statistics of the three pollutants. The difference between the minimum and maximum values of each of the three pollutants was large. This indicates that the pollution emissions of HDDTs in the BTH region is very unbalanced. The standard deviation of PM was the largest which implies that PM pollutants emitted by HDDTs in districts and counties in the BTH region tend to be more dispersed than the other two pollutants.

### 3.2. Spatial Autocorrelation Analysis

There are two kinds of spatial autocorrelation methods: Global Moran’s I and Local Moran’s I. Global Moran’s I was used to measure the spatial autocorrelation characteristics of the entire region to identify the spatial distribution pattern of emissions from HDDTs in the BTH region. The detailed calculation formula is:

(3) $$I=\frac{N\sum_{i=1}^{N}\sum_{j=1}^{N}w_{ij}\left(x_{i}-\bar{X}\right)\left(x_{j}-\bar{X}\right)}{\sum_{i=1}^{N}\sum_{j=1}^{N}w_{ij}\sum_{i=1}^{N}{\left(x_{i}-\bar{X}\right)}^{2}}$$

where I represents the value of Global Moran’s I. $x_{i}$ and $x_{j}$ represent the emission of a pollutant (e.g., NO_X_, PM, SO_2_) from HDDTs in the district and county i and j; $\bar{X}=\frac{1}{N}\sum_{i=1}^{N}x_{i}$ refers to the average emission of a certain pollutant from HDDTs in 200 districts and counties in the BTH region; N refers to the number of districts and counties; here, N = 200; $w_{ij}$ is the spatial weight matrix, where *w_ij_* = 1 if two districts or counties have a common edge or common vertex, otherwise, *w_ij_* = 0. 

Global Moran’s I is an inferential spatial pattern analysis method based on the probability theory. The significance of Global Moran’s I was tested by the standard statistics $Z_{I}$ score and p-value [24]. The expression of $Z_{I}$ is as follows:

(4) $$Z_{I}=\frac{I-E\left(I\right)}{\sqrt{V\left(I\right)}}$$

(5) E(I)=−1/(N−1)

(6) $$V\left(I\right)=E\left(I^{2}\right)-E{\left(I\right)}^{2}$$

where E(I) represents the expected value and V(I) the variance of Global Moran’s I. At 0.1 significance level, $Z_{I}>$ 1.65 indicates positive spatial autocorrelation among the spatial units; $-1.65<Z_{I}<1.65$ indicates that the spatial relationship of the HDDT emissions is not obvious; $Z_{I}<-$1.65 indicates negative spatial autocorrelation among the spatial units. 

Local Moran’s I [42] was used to calculate the degree of correlation of a certain pollutant (such as NO_X_, PM, SO_2_) emitted by HDDTs in each district and county and the neighboring district and county. The expressions for Local Moran’s I are:

(7) $$I_{i}=\frac{\left(x_{i}-\bar{X}\right)}{S^{2}}\overset{N}{\underset{j=1,j\neq i}{\sum }}w_{ij}\left(x_{j}-\bar{X}\right)$$

(8) $$Z_{i}=\frac{\left(x_{i}-\bar{X}\right)}{S}$$

(9) $$w_{z}=\frac{\sum_{j=1,j\neq i}^{N}w_{ij}\left(x_{j}-\bar{X}\right)}{S}$$

where $I_{i}$ indicates the Local Moran’s I value of HDDT emission in the ith district and county of the BTH region. $x_{i}$ and $x_{j}$ represent the emissions of a certain pollutant (such as NO_X_, PM, SO_2_) from HDDTs in i and j districts and counties. $S^{2}$ denotes the variance of emission. $\bar{X}$ denotes the average emission. $w_{ij}$ represents the spatial weight matrix. $I_{i}$ can be divided into two parts: descriptive variable $Z_{i}$ and spatial lag variable $w_{z}$. When $Z_{i}>0$ and $w_{z}>0$, $I_{i}>0$ is a positive correlation indicating that the $x_{i}$ region and $x_{i}$ neighborhood have a high value distribution, consistent with a high–high cluster (HH). When $Z_{i}>0$ and $w_{z}<0$, $I_{i}<0$ is a negative correlation, indicating that the $x_{i}$ area has a high value distribution and the $x_{i}$ neighborhood has a low value distribution, consistent with a high–low outlier (HL). When $Z_{i}<0$ and $w_{z}>0$, $I_{i}<0$ is a negative correlation indicating that the $x_{i}$ region has a low value distribution and the $x_{i}$ neighborhood has a high value distribution, consistent with low–high outlier (LH). When $Z_{i}<0$ and $w_{z}<0$, $I_{i}>0$ is a positive correlation, indicating that the $x_{i}$ region and $x_{i}$ neighborhood have low value distributions, consistent with a low–low cluster (LL).

### 3.3. Regression Analysis

Ordinary Least Square (OLS) is a classical regression model which is widely used to explore the potential impact mechanisms of pollutant emissions. The premise of the model is that dependent variables are independent of each other. The calculation formula is as follows:

(10) Y=Xβ+ε

where Y represents a 200 × 3 column vector, representing the emissions from HDDTs (NO_X_, PM, SO_2_) in 200 districts and counties. X refers to a matrix of 200 × 5 independent variables. β refers to a 5 × 3 vector matrix of regression coefficients. ε refers to a 200 × 3 vector matrix of random interference terms, satisfying Ε(ε)=0, $\mathrm{Cov}\left(\varepsilon_{i},\varepsilon_{i}\right)=\sigma^{2}$, $\mathrm{Cov}\left(\varepsilon_{i},\varepsilon_{j}\right)=0$.

However, existing studies have shown that there are spatial autocorrelation characteristics between vehicle emissions and socioeconomic factors [23,25,26]. Therefore, the results obtained by using OLS without considering spatial effects will be biased or non-optimal. In this study, the spatial econometric model was used to further analyze the potential impact factors of HDDT emissions. Common spatial econometric models include the spatial error model (SEM) and spatial lag model (SLM).

The spatial error model (SEM) was also called the spatial autocorrelation model which indicates that there is spatial autocorrelation in the random error term of the model. It is expressed as follows:

(11) $$y_{ni}=X_{nij}\beta +\lambda W\varepsilon +\mu ,\;\mu ∼N\left(0,\delta^{2}\right)$$

where n=1,…,200 represent the 200 districts and counties in the BTH region. i=1,2,3 represents NO_X_, PM, and SO_2_, respectively. $X_{nij}$(j = 1,…,6) denotes the independent variables of pollutants in each district and county: per capita GDP, population density, urbanization rate, proportion of secondary industry, and proportion of tertiary industry. β denotes the spatial regression coefficient. λ denotes the coefficient of the spatial error term. ε denotes the random error term vector. μ denotes the random error vector obeying a normal distribution.

In the spatial lag model (SLM) or spatial autoregressive model, the dependent variable has a spatial dependence effect and the related influencing factors of the adjacent area have a significant effect on the local dependent variable. It is expressed as follows:

(12) $$y_{ni}=X_{nij}\beta +\rho Wy+\varepsilon$$

where ρ represents the coefficient of the spatial lag factor Wy of the dependent variables. If it is significant, we infer that there is a spatial dependence effect of the HDDT emissions, and its size reflects the intensity of the spatial spillover effect. ε is a random error term.

In practical application, according to the Anselin (2005) standard [43], the suitability of the spatial error model and spatial lag model is decided by comparing the significances of the Lagrange multiplier test statistics LM(error) and LM(lag) of the models. If both are significant, the OLS results are not applicable. Then, the robustness statistics of the spatial error model (LR-LM(error)) and the spatial lag model (LR-LM(lag)) are compared. If LR-LM(error) is significant, the spatial error model is selected. If LR-LM(lag) is significant, the spatial lag model is selected.

### 3.4. Geographical Detector Technique

On the basis of the significant related factors of HDDT emission, this study used the geographical detector technique to further determine the order of strengths of the significant related factors. Unlike the regression model, the geographical detector does not contain a linear hypothesis, and can be used to detect the linear and non-linear correlations between independent variables and dependent variables. The core concept is as follows: if an independent variable has a significant impact on the dependent variable, the spatial distribution pattern of the independent variable and the dependent variable will tend to be the same [31,44]. The q statistic with clear physical meaning is used to express the strength of the correlation between the independent and dependent variables [45], that is, to what extent an independent factor explains the spatial stratified heterogeneity of the dependent variable. The calculation formula is as follows:

(13) $$q=1-\frac{\sum_{h=1}^{H}N_{h}\sigma_{h}^{2}}{N\sigma^{2}}$$

where h is the index of the layer, i.e., the classification or partition of the dependent variable Y or independent variable Χ. $N_{h}$ and N represent the number of elements in the layer h and in the entire region, respectively. $\sigma_{h}^{2}$ and $\sigma^{2}$ represent the variances of the dependent variable Y in the layer h and in the entire region, respectively. H is the number of layers in the entire region. The value range of q is (0,1). The larger the value, the stronger the explanatory power of the independent variable in comparison with that of the dependent variables. In the geographical detector, the independent variables must be the categorical variables. Therefore, we needed to divide the five socioeconomic factors into five categories by using the natural discontinuity method, as shown in Table 4.

## 4. Results

### 4.1. Spatial Distribution Characteristics of Pollutant Emissions from HDDTs in the BTH Region 

#### 4.1.1. Spatial Distribution Pattern of Pollutant Emissions from HDDTs in the BTH Region

**Figure 3:**
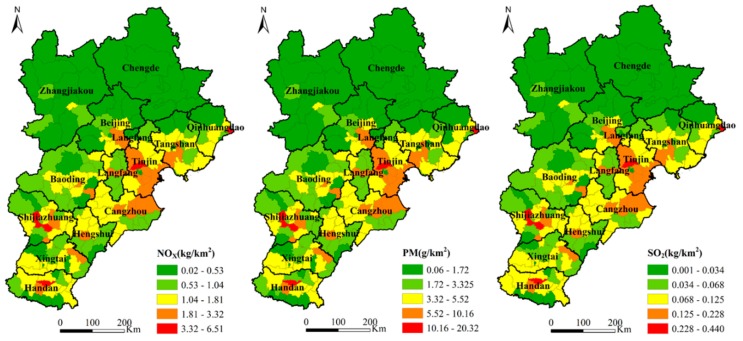
Spatial distribution maps of NO_X_, PM, and SO_2_ emissions from HDDTs in 200 districts and counties in the Beijing–Tianjin–Hebei (BTH) region.

Figure 3 shows the spatial distribution pattern of NO_X_, PM, and SO_2_ emissions per unit area of HDDTs in each district and county in the BTH region. The three pollutants showed similar overall spatial distribution trends, that is, lower in the north, higher in the east, and higher in the south. The maximum and minimum values of each of the three pollutants differed widely. It shows that the HDDT emissions in 200 districts and counties in the BTH region were extremely unbalanced and marked by spatial heterogeneity [46]. The districts and counties with lighter emissions were mainly concentrated in Chengde and Zhangjiakou in the northern BTH region. The reason is that Chengde and Zhangjiakou have limited industrial development [32], and the terrain type is mainly plateau and mountainous areas which are important pioneer ecological civilization demonstration zones and present an important ecological security barrier for the BTH region. Therefore, the regional transportation demand for HDDTs is relatively small. Heavily polluted districts and counties are located in Tianjin, Cangzhou, Tangshan, Shijiazhuang, and Handan. Among them, Tianjin is located in the coastal area with unique location advantages. Long- and medium-distance transportation of port containers for coal and other bulk cargo aggravate the emission of pollutants from HDDTs. Cangzhou, Tangshan, Shijiazhuang, and Handan have strong industrial bases with heavy industry as the main industrial structure [47]. The transportation of industrial raw materials depends on the road transportation dominated by HDDTs, resulting in relatively high HDDT emissions in these areas.

#### 4.1.2. Spatial Autocorrelation Characteristics of HDDT Emissions in the BTH Region

The global spatial dependence of the three pollutants was explored using Global Moran’s I. The results are shown in Table 5. NO_X_, PM, and SO_2_ had significant spatial dependences. The value of Global Moran’s I of NO_X_, PM, and SO_2_ were 0.2808, 0.2775, and 0.2851, respectively. The Z score of the three pollutants was more than 2.58, and the *p*-values were less than 0.01. This shows that there are significant positive correlations among the emissions of the three pollutants by HDDTs in the 200 districts and counties in the BTH region. Therefore, the areas with high emissions of NO_X_, PM, and SO_2_ also had high emissions of the same pollutants in their surrounding districts and counties, and the districts and counties with low emissions of NO_X_, PM, and SO_2_ had low emissions of the same pollutants in their surrounding districts and counties.

**Figure 4:**
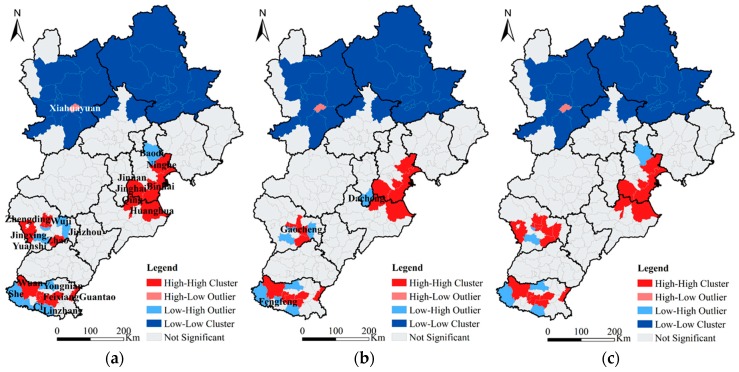
Local spatial autocorrelation results of NO_X_ (**a**), PM (**b**), and SO_2_ (**c**) pollutants.

Furthermore, the local spatial dependence of HDDT emissions was explored by using Local Moran’s I. The results are shown in Figure 4. The spatial distributions of NO_X_, PM, and SO_2_ were similar. High–high clusters are marked as hot spots, mainly distributed in Tianjin, Cangzhou, Shijiazhuang, and Handan. Low–low clusters are marked as cold point areas, mainly concentrated in Zhangjiakou and Chengde. The number of hot spots and cold spots accounted for the largest proportion of the total number of significant districts and counties. The only district with a high–low outlier was Xiahuayuan which shows that the Xiahuayuan District had an abnormally high concentration of hot spots relative to the surrounding cold point areas. The reason is that Xiahuayuan District is rich in mineral resources and is the main coal-producing base of Zhangjiakou. It is also an important transportation channel for coal in Shanxi and Inner Mongolia. The transportation demands and traffic volumes of HDDTs are relatively large, resulting in higher pollution emissions. The Low-High Outliers are mainly distributed near the hot spots, such as Lubei District and Guzhi District of Tangshan, Luancheng District of Shijiazhuang, and around the hot spots in Handan. Combining Global Moran’s I and Local Moran’s I, it can be seen that the pollution emission of HDDTs in the BTH region present the characteristics of regional agglomeration. This emphasizes the importance of interregional collaborative governance for effective emission control of HDDTs.

### 4.2. Analysis of Related Factors of HDDT Emissions in the BTH Region

#### 4.2.1. Significances and Directions of Related Factors

In this study, regression analysis was used to detect the significances and directions of the related factors of heavy-duty diesel vehicle emissions. The results of three models are shown in Table 6. The *R*^2^ of ordinary least squares (OLS) was equal to 0.61, and the *R*^2^ of the spatial error model and spatial lag model were 0.66 and 0.67, respectively. This shows that the fitting effect of the model with spatial effect was enhanced. The OLS results were used to determine the spatial econometric model that should be used. It was found that the Lagrange statistic Lm (lag) and Lm (error) of the spatial lag model and the spatial error model were significant, whereas the robust statistic LR-LM (lag) of the spatial lag model was more significant than the robust statistic LR-LM (error) of the spatial error model; therefore, the spatial lag model was chosen.

The results of the spatial lag model show that the urbanization rate, proportion of secondary industries, and proportion of tertiary industries were significantly correlated with NO_X_, PM, and SO_2_ emissions from HDDTs. Population density and per capita GDP did not pass the significance test. Among the significant factors, the urbanization rate and the proportion of secondary industries were positively correlated with the HDDT emissions, whereas the proportion of tertiary industries was negatively correlated with the HDDT emissions. The urbanization rate tends to aggravate emissions from HDDTs, which is consistent with the existing research results. Han et al. [48] believed that with increases in the urbanization rate, the concentration of PM_2.5_ in Chinese cities also increases. Frequent human activities lead to increased air pollution [49,50]. The results show that the relationship between the urbanization rate and HDDT emission has not passed the top of the inverted U-shape of the Environmental Kuznets Curve. The proportion of secondary industries was positively correlated with the HDDT emissions. With an increase in the proportion of secondary industries, the transportation requirement for raw materials and products expands, and the demand for HDDTs for transportation increases, which aggravates the pollution caused by HDDT emissions. There was a significant negative correlation between the proportion of tertiary industries and HDDT emission. The reason is that tertiary industries, which mostly comprise information technology and service industries belong to the non-material production sector; they have a high degree of resource integration and relatively low energy consumption. Therefore, the demand for HDDTs for transportation and thereby the HDDT emission decrease which shows that a higher proportion of tertiary industries can restrain HDDT pollution. In addition, the spatial lag model calculates the endogenous interaction effect (*W* × ln*Y*) of the pollutants which shows that there is a significant spatial spillover effect in pollutant emissions. *W* × lnY passed the significance test of 1%. It indicates that when other variables remain unchanged, for every 1% increase in NO_X_, PM, and SO_2_ emissions from HDDTs in the surrounding districts and counties, NO_X_, PM, and SO_2_ emissions from local HDDTs will increase by 0.39%.

#### 4.2.2. Strengths of Significant Related Factors

**Figure 5:**
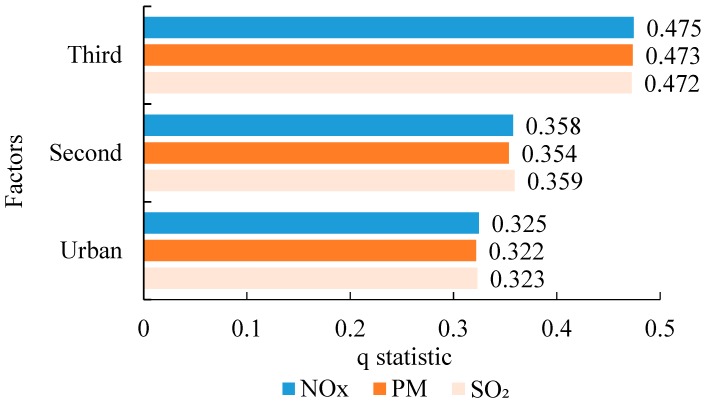
Results of the geographical detector technique for NO_X_, PM, and SO_2_.

After determining the significant related factors, this study used the geographical detector technique to further quantify the strength of the significant related factors. The results are shown in Figure 5. The q statistic was between 0.3217 and 0.4745. The strengths of the proportion of tertiary industries, proportion of secondary industries, and urbanization rate on NO_X_, PM, and SO_2_ were similar. The order of the effect intensities of the significant correlation factors is as follows: proportion of tertiary industries > proportion of secondary industries > urbanization rate. The proportion of tertiary industries had the strongest inhibitory effect of up to 47% on heavy-duty diesel vehicle pollution emissions, whereas the proportion of secondary industries had more than a 35% aggravating effect on heavy-duty diesel vehicle emissions. This shows that the industrial structure has a significant impact on HDDT emissions. The results are significant for pollution control of heavy diesel vehicles. Measures to reduce the pollutants emitted by heavy diesel vehicles should focus on transitioning from secondary industries to tertiary industries. Without affecting the economic growth, we should realize the eco-friendly transition of industrial production from extensive to intensive. In addition, the urbanization rate and haze pollutants emitted by HDDTs exhibit a significant trend of synchronous growth. This shows that the relationship among them still lies in the left half of the Environmental Kuznets Curve, and the urbanization rate of the districts and counties in the BTH region has not passed the vertex of the Environmental Kuznets Curve. The reason is that with increases in the urbanization rate, there is a shift in population from rural areas to cities and towns, and the type of occupation changes from farming to employment in energy-intensive heavy industries. The transportation of industrial raw materials, industrial products, and other bulk goods relies on road transportation mainly by HDDTs which increases HDDT emissions. In the future, we should pay more attention to environmental protection while ensuring economic development by developing the technical means to promote energy recycling and improving the transport efficiency of HDDTs, so as to reduce HDDT emissions.

## 5. Discussion

Vehicle pollution has become an important source of urban environmental pollution in China. Heavy-duty diesel trucks are the main contributors of NO_X_ and PM and have become the primary source of vehicle emissions resulting in haze. Therefore, a thorough understanding of the spatial distribution characteristics and potential influencing factors of heavy-duty diesel vehicle emissions can provide reliable data support for effective control of diesel vehicle pollution control and assist environmental protection and related departments in formulating scientific emission reduction strategies.

Firstly, the spatial distribution of pollutants from HDDTs in the BTH region is extremely unbalanced, i.e., low in the north and high in the east and south. High emission values are concentrated in Tianjin, Tangshan, Cangzhou, Shijiazhuang, and Handan. Therefore, while formulating emission reduction measures for HDDTs, the relevant departments should fully consider the balance between regional economic development and environmental pollution. For example, the financial subsidies for upgrading HDDTs and acquiring new energy vehicles can be appropriately shifted to the eastern and southern parts of the BTH region. Tianjin, Tangshan, Cangzhou, Shijiazhuang, and Handan should be listed as key cities for prevention and control of HDDT emissions.

Secondly, the effect of local spatial agglomeration is obvious, and the diffusion of pollutants among regions leads to significant influence of local pollutants on the neighboring regions. This shows that the HDDT emissions in the BTH region show the trend of regional integration, and it is necessary to further strengthen the synergistic prevention and control of regional pollution in the BTH region. Specifically, an ecological compensation mechanism can be adopted to rationalize the cost of pollution control for HDDTs.

Thirdly, the restraining effect of the proportion of tertiary industries is the strongest, followed by the additive effect of secondary industries with heavy industry as the main constituent. This indicates that in the future, the BTH region should accelerate the upgrading of the industrial structure, realize the transition from heavy industry to tertiary industry with highly integrated resources, and fundamentally improve the deployment efficiency of heavy diesel vehicles, thereby reducing the emissions of NO_X_, PM, and SO_2_ from HDDTs. The urbanization rate plays a significant role in promoting HDDT emissions. It shows that the relationship between the socioeconomic development of districts and counties in the BTH region and the HDDT emissions has not passed the vertex of the inverted U-shape of the Environmental Kuznets Curve. Therefore, while pursuing economic development, we should enhance the awareness regarding environment protection, improve the technical measures employed for it, and increase the related investment. 

## 6. Conclusions

The existing research on vehicle pollution emission emphasizes the dominant role of HDDT emissions. Because HDDT emissions have not been adequately studied heretofore, we performed a detailed and systematic study of NO_X_, PM, and SO_2_ emissions from HDDTs with the BTH region as an example study area. 

The technical method involving the spatial characteristics and correlation analysis of HDDT emissions provides a reference for other regions in the world where pollution from HDDTs is a grave concern. Firstly, this study explored the spatial characteristics of HDDT emissions, including the spatial distribution pattern and spatial autocorrelation characteristics, in 200 districts and counties in the BTH region. Secondly, we explored the related socioeconomic factors that influence the assessed spatial characteristics of HDDTs from the perspective of social economy. The main steps of the correlation analysis were as follows: (1) the spatial econometric model was used to calculate the significances and directions of the correlation factors; (2) a geographical detector technique was used to quantify the order of the effect intensities of the significant related factors. The results showed that there are significant spatial heterogeneity and spatial autocorrelations among the factors influencing the HDDT emissions in 200 districts and counties of the BTH region. The emission hot spots are concentrated in the coastal areas and counties with a strong industrial base, where the demand for HDDTs for transportation is high. The low emission values are mainly distributed in the pioneer ecological civilization demonstration zones in the north BTH region, where the industrial development is limited and the demands for HDDTs for transportation is low. The significant socioeconomic factors that cause this spatial distribution are the proportion of tertiary industries, proportion of secondary industries, and the urbanization rate. Among them, the proportion of tertiary industries has a negative correlation with the HDDT emissions, and the proportion of secondary industries and the urbanization rate have positive correlations with the HDDT emissions. The order of effect intensities of the factors are as follows: proportion of tertiary industries > proportion of secondary industries > urbanization rate. The results of this study can guide environmental protection departments to formulate targeted emission reduction measures for HDDTs.

Owing to the non-availability of data, this study obtained the trajectory data of the BTH region for only one month. However, previous studies have shown that the seasonal variation of pollution is obvious [51]. Therefore, future research will collect trajectory data from other months and consider the time-varying characteristics of the HDDT emissions. In addition, from the perspective of social economy, this study explored the interaction mechanism between HDDT emissions and socioeconomic indicators. In the future, natural factors such as slope, wind direction, wind speed, and temperature can be added to the research. Based on the social economy and natural factors, the related factors of HDDT emission can be analyzed to provide a basis for decision making for relevant departments to formulate scientific and reasonable emission reduction measures.

## Figures and Tables

**Table 1:** Activity data and specifications of heavy-duty diesel trucks (HDDTs) at different sampling intervals.

Time	Longitude	Latitude	Vehicle ID	Speed (km/h)	Tonnage (t)	Emission Standards
15 April 2018 00:01:29	114.793419	37.773788	101203	67.24	31.0	Euro IV
15 April 2018 00:01:44	118.388985	39.673519	102576	44.37	24.8	Euro III
15 April 2018 00:01:59	117.524101	35.917999	257364	59.82	20.5	Euro V
15 April 2018 00:02:39	114.101501	36.595001	432576	87.83	15.9	Euro IV
…	…	…	…	…	…	…

**Table 2:** Statistical data of explanatory variables. The symbol predictions refer to the expected direction of change of the variables affecting the emissions.

Explanatory Variable	Abbreviation	Symbol Predictions	Minimum	Maximum	Mean	SD	Moran’s I
Per capital GDP (ten thousand)	GDP	+	1.22	32.14	5.00	0.29	0.45 ***
Population density (people/km^2^)	People	+	41.77	41,967	1986.92	355.76	0.53 ***
Urbanization rate (%)	Urban	+	15.78	100.00	57.63	1.49	0.43 ***
Proportion of secondary industries (%)	Second	+	1.43	68.63	41.20	1.03	0.27 ***
Proportion of tertiary industries (%)	Third	−	24.36	98.57	47.43	1.19	0.44 ***

GDP: gross domestic product. *** denote that the values passed the significance tests of 1%. SD: standard deviation.

**Table 3:** Detailed statistics for heavy-duty diesel vehicle emission inventories.

Pollutant	Unit	Minimum	Maximum	Average	SD
NO_X_	kg/km^2^	0.0207	6.5042	1.1272	0.9096
PM	g/km^2^	0.0625	20.3228	3.4235	2.7907
SO_2_	kg/km^2^	0.0014	0.4396	0.0774	0.0619

**Table 4:** Classifications of independent variables.

Independent Variables	Classification 1	Classification 2	Classification 3	Classification 4	Classification 5
lnGDP	≤0.8	0.8–1.2	1.2–1.7	1.7–2.4	2.4–3.5
lnpeople	≤5.0	5.0–6.0	6.0–7.0	7.0–8.3	8.3–10.7
lnurban	≤3.5	3.5–3.8	3.8–4.0	4.0–4.3	4.3–4.7
lnsecond	≤2.2	2.2–3.2	3.2–3.6	3.6–3.9	3.9–4.3
lnthird	≤3.3	3.3–3.4	3.4–3.5	3.5–3.6	3.6–3.9

**Table 5:** Global Moran’s I.

Pollutant	Moran’s I	Z Score	*p*-Value
NO_X_	0.2808	6.6048	<0.01
PM	0.2775	6.5398	<0.01
SO_2_	0.2851	6.6985	<0.01

**Table 6:** Regression model results of NO_X_, PM, and SO_2_. OLS: ordinary least squares; SLM: spatial lag model; SEM: spatial error model.

Pollutant	Variable	OLS	SLM	SEM
	CONSTANT	4.5533 *	29.7975 ***	35.952 ***
	lnGDP	0.0983	0.0957	0.1650
	lnpeople	0.0002	0.0354	−0.1931*
NO_X_	lnurban	1.2703 ***	1.4544 ***	1.8731 ***
	lnsecond	1.1472 ***	0.9339 ***	0.8451 ***
	lnthird	−8.7645 ***	−9.1260 ***	−9.4088 **
	W × ln NO_X_		0.3916 ***	
	CONSTANT	28.3899 ***	26.4641 ***	30.3236 ***
	lnGDP	0.0986	0.0955	0.1683
	lnpeople	0.0021	0.0368	−0.1918 *
PM	lnurban	1.3109 ***	1.4835 ***	1.8983 ***
	lnsecond	1.1390 ***	0.9292 ***	0.8392 ***
	lnthird	−8.8786 ***	−9.2130 ***	−9.4854 ***
	W × ln PM		0.3924 ***	
	CONSTANT	37.9129 ***	32.0907 ***	39.9361 ***
	lnGDP	0.1058	0.1013	0.1705
	lnpeople	−0.0010	0.0341	−0.1933 *
SO_2_	lnurban	1.2540 ***	1.4376 ***	1.8598 ***
	lnsecond	1.1369 ***	0.9254 ***	0.8360 ***
	lnthird	−8.6586 ***	−9.0186 ***	−9.3125 ***
	W × ln SO_2_		0.3913 ***	

*, **, and *** denote values that pass the significance tests of 10%, 5%, and 1%, respectively.
